# Initial Crystallization Effects in Coarse-Grained Polyethylene Systems after Uni- and Biaxial Stretching in Blow-Molding Cooling Scenarios

**DOI:** 10.3390/polym14235144

**Published:** 2022-11-26

**Authors:** Dirk Grommes, Martin R. Schenk, Olaf Bruch, Dirk Reith

**Affiliations:** 1 Institute of Technology, Resource and Energy-Efficient Engineering (TREE), Bonn-Rhein-Sieg University of Applied Sciences, Grantham-Allee 20, 53757 Sankt Augustin, Germany; 2 Dr. Reinold Hagen Stiftung, Kautexstrasse 53, 53229 Bonn, Germany; 3 Fraunhofer Institute for Algorithms and Scientific Computing (SCAI), Schloss Birlinghoven, 53754 Sankt Augustin, Germany

**Keywords:** mesoscale coarse-graining, polyethylene, biaxial stretching, relaxation, crystallization, local chain orientation

## Abstract

This study investigates the initial stage of the thermo-mechanical crystallization behavior for uni- and biaxially stretched polyethylene. The models are based on a mesoscale molecular dynamics approach. We take constraints that occur in real-life polymer processing into account, especially with respect to the blowing stage of the extrusion blow-molding process. For this purpose, we deform our systems using a wide range of stretching levels before they are quenched. We discuss the effects of the stretching procedures on the micro-mechanical state of the systems, characterized by entanglement behavior and nematic ordering of chain segments. For the cooling stage, we use two different approaches which allow for free or hindered shrinkage, respectively. During cooling, crystallization kinetics are monitored: We precisely evaluate how the interplay of chain length, temperature, local entanglements and orientation of chain segments influence crystallization behavior. Our models reveal that the main stretching direction dominates microscopic states of the different systems. We are able to show that crystallization mainly depends on the (dis-)entanglement behavior. Nematic ordering plays a secondary role.

## 1. Introduction

Semi-crystalline polymers are widely used in industrial and consumer-related applications [1]. Their individual performance characteristics give polymers versatile usage options. During polymer processing, their properties change, primarily due to orientation and crystallization processes under the occurrence of flow fields [2,3,4,5] or stretching processes [6,7]. These varying changes put significant difficulties into the design process of plastic parts. Computer-aided engineering (CAE) has become increasingly important for optimal product design over the past several years. Simulation-based product tests using the method of finite elements (FE) have already been state of the art for decades [7,8,9,10,11,12]. In the field of blow-molding, such simulation models already use input data concerning biaxial stretching during processing [7,8,10,11]. Performing the necessary experiments to characterize stretch dependent material data is associated with high costs. Consequently, simulation models often use a coarse process-dependent material description only [7,8,10,11]. A way to overcome that limitation is using multiscale molecular dynamics (MD) simulation methods [13,14]: Modelling a specific polymer on the microscale enables the determination of material parameters needed for the simulations on a mesoscopic or even macroscopic scale.

In this work, we investigate the microscopic state of polyethylene after different uni- and biaxial stretching processes. We mainly determine relationships between the micro-mechanical state after stretching and the following initial crystallization behavior. We use other loading conditions, which resemble real-life processing conditions. We stretch and cool our systems under two different approaches that both typically occur in real-life processing: (1) “fixed conditions”, where the box size (‘’the part shape”) is fixed in every direction of space to mimic the effect of a mold constraint. Only after cooling is finished (and the part is demolded), the box (‘’the part”) is allowed to change its shape and size. (2) “free conditions”, where the system (‘’the real part”) can deform immediately after stretching. We mainly concentrate on investigating fixed boundary conditions during cooling as we want to conserve the micro-mechanical state after stretching and precisely monitor the resulting crystallization behavior. Furthermore, the fixed conditions resemble real-life plastics processing boundaries closely, especially with our focus on extrusion blow-molding and similar processes such as deep drawing or film blowing. By using these specific boundary conditions, we tie to the proposed ideas of Chandran et al. [15], who encourage the consideration of real-life processing conditions within molecular dynamics simulations.

During stretching of the melt, flow-enhanced nucleation (FEN) occurs, as described by the theory of Flory’s conformational entropy reduction model (CERM) [16]. Different MD studies discussing the crystallization of priorly deformed polyethylene systems are found in [17,18,19,20,21,22,23]. These studies focus on uniaxially stretched or sheared systems only. In our previous work [24], we investigated uniaxially stretched polyethylene systems’ crystallization and relaxation behavior at one specific level of stretching $\lambda_{\mathrm{uni}}=6$. As real-life processes usually introduce biaxial stretching to the processed melt, drastically changing trends of chain straightening and orientation are likely to occur [25]. In the work of [25], different biaxial levels of stretching for a coarse-grained poly(vinyl alcohol) (PVA) are investigated. They define the level of biaxial stretching by the multiplication of the two planar stretch ratios. For all investigated systems, their total level of biaxial stretching is constant at $\lambda_{\mathrm{biaxial}}=9$. They report a significant influence of the different stretch ratios on orientation and crystallization behavior. Recently, Zhang et al. [26] investigated uni- and biaxially stretched polyethylene systems using MD methods. They focused on the influence of different stretching approaches on stress–strain properties and failure behavior. For the influence of various force fields on the crystallization behavior, an overview of united atom (UA) models is given by Hagita et al. [27].

## 2. Simulation Methodology

### 2.1. Force Field

We use a coarse-grained polyethylene force field [28] which we previously utilized to simulate tensile tests [29] and relaxation effects in uniaxially stretched systems [24]. It was shown that it is suitable for evaluating entanglement, orientation and crystallization effects that drive the behavior of polyethylene. Therefore it is an ideal starting point for the investigation of strongly biaxially stretched systems. The bonded interaction and Lennard Jones (LJ) parameters for CG polyethylene description are presented in Table 1. LJ parameters [28] are optimized to have good agreement with experimental density and heat of vaporization. For more accuracy [28], defines the LJ parameters depending on the particle position (end or middle position in the chain). Therefore two types of CG beads (CG_mid_, CG3_end_) are defined. The first- and second-neighbor beads are excluded from the non-bonded interactions. Additionally, there is a third-neighbor LJ interaction with different parameters. The cut-off distance $r_{c}$ is taken as 2.5 times the value of σ of the middle bead. All non-bonded parameters are summarized in Table 1.

Special attention is paid to the proper equilibration of polymer melts as this is a highly non-trivial task. We apply the equilibration procedure from Moreira et al. [30] and Auhl et al. [31]. Our previous work shows that [29] we can adopt that procedure for equilibrating our chemically specific force field. By verifying the static melt structure factor and the final systems’ mean square internal distance, we ensure that these are well equilibrated. The results of the equilibration are found in [29]. We equilibrate and further investigate four different system sizes with various numbers of chains *M* and chain length *N* (M×N: 1000×500, 100×1000, 500×1000, 250×2000). Our equilibrated systems have density of the amorphous phase at 293 K ($\rho_{\mathrm{amorph},293K}$), melt density ($\rho_{500K}$), coefficient of thermal expansion (CTE), glass transition temperature ($T_{g}$) and crystallization onset temperature ($T_{c,\mathrm{on}}$) in good agreement with experimental results [24].

### 2.2. Simulation Procedure

Molecular dynamics simulations are performed using the ESPResSo++ package [32,33]. Starting with the equilibrated systems, our simulation procedure consists of two steps: (1) stretching of the samples of amorphous melt, (2) quenching of the samples to a specific temperature at two different conditions.

In the first step, we continuously stretch the systems in the melt state at 500 K at an initial strain rate of $1\;\cdot \;10^{8}\;s^{-1}$. The selected strain rate ensures strong orientations of chain segments without over-pronounced disentanglement of chains. We stretch the systems up to different uni- and biaxial levels: In the case of uniaxial stretching, we stretch the systems up to a level $\lambda_{\mathrm{uni}}=6$. Additionally, we use different biaxial levels of stretching ($\lambda_{x}$-$\lambda_{y}$) ranging from 2-2, 3-1.5, 3-2, 4-2 to 3-3. The stretching is performed under usage of the Berendsen barostat for the transversal directions ($\tau_{\mathrm{baro}}=10\;\mathrm{ps}$), Berendsen thermostat ($\tau_{\mathrm{thermo}}=1\;\mathrm{ps}$) and periodic boundary conditions (rectangular box).

Cooling the samples while releasing the tensile strain is done in two distinct ways, mimicking other real-life processing conditions. The first option is choosing “fixed” boundary conditions, which do not allow the previously stretched melt to contract after the final levels of stretching are attained. This allows for the internal relaxation of stress while the system cannot change its size and shape. Simultaneously, we cool the systems down to the target temperature (293.15 K) within a time frame of 10 ns by using the Berendsen thermostat ($\tau_{\mathrm{thermo}}=1\;\mathrm{ps}$). The second option for the cooling procedure is a “free” boundary condition: We here allow the box dimensions to change during cooling. This is done by using an anisotropic Berendsen barostat ($\tau_{\mathrm{baro}}=1000\;\mathrm{ps}$) and setting the pressure to 1 bar in every direction of space. The integration time step for all simulation steps is set to 4 fs.

### 2.3. Evaluation of the Microscopic Structure

In order to achieve comparability of different uni- and biaxial stretchings, we define the planar level of stretching $\lambda_{\mathrm{planar}}$. We here use the ratio of the initial and final diagonal length of the simulation boxes within the stretching plane (x-y plane). From that, the planar level of stretching is defined as $\lambda_{\mathrm{planar}}=\sqrt{l_{\mathrm{final},x}^{2}+l_{\mathrm{final},y}^{2}}/\sqrt{l_{\mathrm{init},x}^{2}+l_{\mathrm{init},y}^{2}}$, where $l_{\mathrm{final}}$ is the box dimension in the corresponding stretching direction at the end of the stretching, $l_{\mathrm{initial}}$ represents initial box dimensions. Our definition is useful as it makes resulting data points across different uni- and biaxial stretchings collapse on a single line with respect to a wide range of observables. Hence, we prefer that definition over a multiplication of the two stretch ratios as, e.g., used in [25].

We introduce the nematic order parameter to evaluate the local ordering of chain segments. For this purpose, we use the unit bond vectors ${\vec{e}}_{i}$, which connect consecutive beads that belong to the same chain. The related nematic tensor can be expressed as

(1) $$Q_{\alpha \beta }=\frac{1}{N_{p}}\sum_{i=1}^{N_{p}}\left(\frac{3}{2}e_{i\alpha }e_{i\beta }-\frac{1}{2}\delta_{\alpha \beta }\right)$$

where $N_{p}$ is number of evaluated bonds and α,β∈(x,y,z). The nematic order parameter *S* is the largest eigenvalue of this tensor. The local nematic order parameter $S_{\mathrm{local}}$ is determined for every bead *i* by evaluating all bonds found within a cutoff distance $r_{\mathrm{cut}}=2\sigma$ from the *i*th bead. For the entire system, we define ${\bar{S}}_{\mathrm{local}}$ as the mean value over all beads. The analysis is performed using the corresponding implementation in the Freud package [34].

Based on the formulation of the nematic order parameter, we calculate the uniaxial orientation factor $\delta_{\mathrm{uni}}$ as follows: (a) determination of the local bond vector orientation factor $\delta_{x}$ in longitudinal stretching direction as already introduced in [24]; (b) determination of the corresponding orientation factor $\delta_{y}$ in perpendicular direction; (c) the uniaxial orientation factor is $\delta_{\mathrm{uni}}=(\left|\delta x-\delta y\right|)/1.5$. This definition gives an estimate to what extent systems are uniaxially ($\delta_{\mathrm{uni}}\to 1$) or (equi-)biaxially ($\delta_{\mathrm{uni}}\to 0$) oriented, respectively.

Entanglements of chains are evaluated by the primitive path analysis (PPA), according to Everaers et al. [35], based on the assumptions of the tube model [36,37,38]. From the PPA, the entanglement length $N_{e}$ according to [35] is:

(2) $$N_{e}=\frac{a_{\mathrm{pp}}}{\langle b_{\mathrm{pp}}\rangle }=\left(N-1\right)\frac{\langle R_{\mathrm{ee}}^{2}\rangle }{{\langle L_{\mathrm{pp}}\rangle }^{2}}$$

where $\langle L_{\mathrm{pp}}\rangle =\left(N-1\right)\langle b_{\mathrm{pp}}\rangle$ is the length of the primitive path (PP), $\langle b_{\mathrm{pp}}\rangle$ is the PP bond length, $a_{\mathrm{pp}}$ the tube radius, which is defined as $a_{\mathrm{pp}}=\langle R_{\mathrm{ee}}^{2}\rangle /\langle L_{\mathrm{pp}}\rangle$. $\langle R_{\mathrm{ee}}^{2}\rangle$ is the mean squared end-to-end distance of the chains. For our evaluations, we define the number of entanglements *Z* per chain as the ratio $N/N_{e}$ (cf. [39]). This relation allows for an estimate of how entangled the systems with different chain lengths are. Further details concerning the use of the PPA with respect to our systems are given in [24].

The level of crystallinity throughout this manuscript is calculated based upon our microscopic definition as reported in [24]:

(1) The current bead *i* is closer to a neighboring bead *j* than $0.975\;\cdot \;2^{1/6}\sigma$. Bonded first neighboring beads are excluded here;

(2) The orientation factor $\delta_{\mathrm{cryst}}$ between vectors ${\vec{V}}_{i-1,i+1}$ and ${\vec{V}}_{j-1,j+1}$ (vectors between first neighboring beads of bead *i* and bead *j*) according to:

(3) $$\delta_{\mathrm{cryst}}=\frac{3}{2}{\left(\frac{\left|{\vec{V}}_{i-1,i+1}\;\cdot \;{\vec{V}}_{j-1,j+1}\right|}{\left|{\vec{V}}_{i-1,i+1}\right|\;\cdot \;\left|{\vec{V}}_{j-1,j+1}\right|}\right)}^{2}-\frac{1}{2}$$

is larger than 0.9;

(3) We define the microscopic crystal stem length $n_{\mathrm{stem}}$ as the number of consecutive beads within a chain that fulfil criteria (1) and (2). All beads that belong to stems with $n_{\mathrm{stem}}\geq 3$ are regarded as being in a crystalline state;

(4) By counting the number of crystalline beads $N_{\mathrm{cb}}$ according to (3), we determine the degree of crystallinity $x_{\mathrm{cryst},\mathrm{micro}}$ by dividing the number of crystalline beads $N_{\mathrm{cb}}$ by the number of total beads $N_{\mathrm{total}}$.

## 3. Results

In the following subsections, we describe the effects that occur in antecedently stretched polyethylene systems. We start by describing the system behavior at the end of the individual stretching procedures. Subsequently, we discuss the resulting states after cooling of the stretched systems to 293.15 K. We mainly focus on evaluating results attained under the usage of fixed conditions. Additionally, we compare the influence of fixed and free conditions on the resulting formation of initial crystalline structures.

### 3.1. System State after Stretching

**Figure 1:**
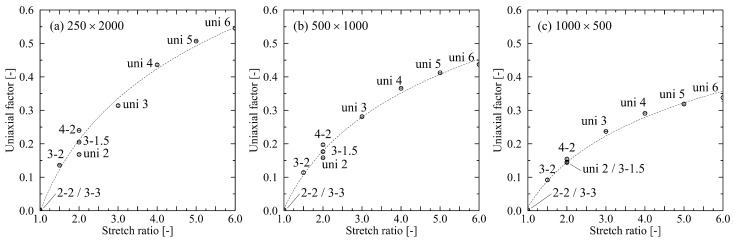
Uniaxial factor for different levels of uni- and biaxial stretching. All systems are stretched at a temperature of 500 K. (**a**) system size: 250×2000, (**b**) system size: 500×1000, (**c**) system size: 1000×500. The dotted line in each graph represents a logarithmic fit and is a guide to the eye only. Inserts denote the levels of stretching for each data point. Error bars are smaller than symbols.

Global inspection of systems shows that the density of the systems after stretching is close to the initial equilibrated system ($0.746\;g/{\mathrm{cm}}^{3}$). Density only decreases after stretching, due to the formation of voids, in the case of strong stretching of systems with long chain length. Involved systems are the 500×1000 (stretching level 3-3: $0.719\;g/{\mathrm{cm}}^{3}$) and the 250×2000 systems (stretching level 3-2: $0.685\;g/{\mathrm{cm}}^{3}$; 4-2: $0.613\;g/{\mathrm{cm}}^{3}$; 3-3: $0.551\;g/{\mathrm{cm}}^{3}$). Deformation of systems to specific degrees of stretching leads to distinctively different microstates. We first evaluate the orientation state by use of our uniaxial factor definition. Figure 1 reveals that the general relation between stretch ratio and level of uniaxial orientation is non-linear for all investigated chain lengths. For the equi-biaxially stretched systems (2-2; 3-3), the uniaxial factor meets expectations by giving almost zero value. Thus, these systems have an equal distribution of orientations in both stretching directions. On the other end of the investigated scale, the uni 6 system shows strong uniaxial orientation, which is clearly reasonable. For a stretch ratio of 2, Figure 1 indicates different orientation states for the investigated systems (uni 2; 3-1.5; 4-2). Despite the same stretch ratio, an increase in the planar level of stretching introduces a more uniaxial orientation of chain segments in the direction of the larger stretch level. This effect is more pronounced for longer chain lengths. Generally, the level of uniaxial orientation increases with increasing chain length.

**Figure 2:**
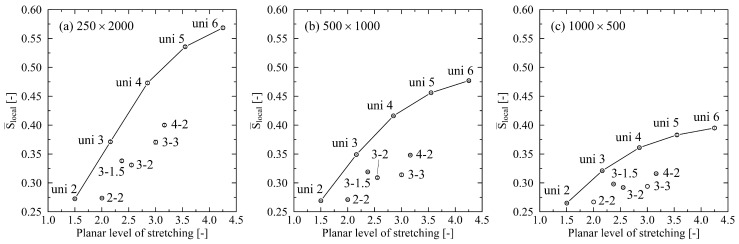
Nematic order parameter for different levels of uni- and biaxial stretching plotted over the planar level of stretching. All systems are stretched at a temperature of 500 K. (**a**) system size: 250×2000, (**b**) system size: 500×1000, (**c**) system size: 1000×500. The data points for the uniaxially stretched systems are connected as a guide to the eye. Inserts denote the levels of stretching for each data point. Error bars are smaller than symbols.

For the evaluation of local chain ordering, we use the nematic order parameter ${\bar{S}}_{\mathrm{local}}$. From Figure 2, it becomes clear that in the case of pure uniaxial stretching, an increasing level of stretching is connected to an increasing level of local ordering. Systems with larger chain lengths have significantly more local ordering than systems with shorter chain lengths. As already stated in [24], this indicates that shorter (thus less entangled) chains tend to be pulled apart as a whole during stretching. Interestingly, biaxial stretching has a negative impact on local ordering, especially if low levels of stretching are used for the minor stretching direction. By comparing systems that are stretched by a level of 3 in the main direction, this effect is obvious: The level of local ordering decreases starting from the uni 3 over the 3-1.5 to the 3-2 system. This indicates a disturbing effect of biaxial stretching compared to pure uniaxial stretching for local alignment of chain segments. This observation holds for all investigated chain lengths (Figure 2a–c). Only in the case of the 250×2000 system at a large biaxial stretching factor of 3-3 the local chain ordering is in the range of the pure uniaxially stretched system (uni 3).

**Figure 3:**
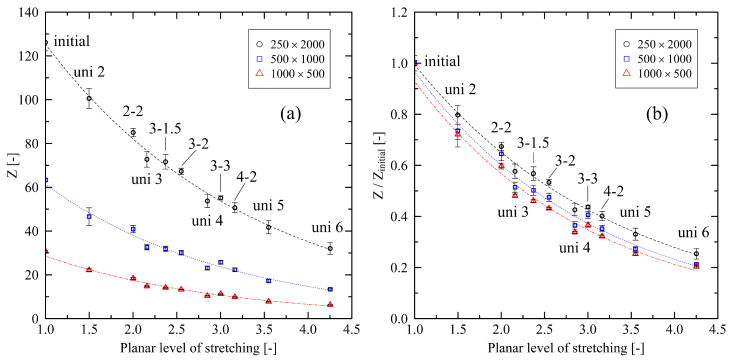
(**a**) Number of entanglements per chain *Z* plotted over the planar level of stretching for different system sizes (M×N). (**b**) Same data shown as normalized plot to the corresponding initial value of *Z*. Inserts denote the levels of stretching for the corresponding data points. The dotted lines represent exponential fits for each system size. They are a guide to the eye only.

Furthermore, we compare the number of entanglements per chain *Z* with the planar degree of stretching. Figure 3 shows that an increasing level of uniaxial stretching leads to a rapid decrease in the number of entanglements. In the case of biaxial stretching, Figure 3 reveals that the main stretching direction dominates disentanglement behavior. The systems that were stretched by a factor of 3 in the major stretching direction (uni 3; 3-1.5; 3-2) in particular demonstrate this effect: Starting at the uni 3 system, additional biaxial stretching leads to a mild decrease in the number of entanglements only. A strong level of stretching of 3-3 is needed to induce a larger decrease in the number of entanglements. Moreover, the number of entanglements for the uni 4 and 4-2 systems are on a very close level. However, the biaxially stretched system is clearly deformed to a higher extent than the uniaxial system. From Figure 3, it is obvious that these findings are valid for all investigated chain lengths. Nevertheless, due to the lower number of entanglements per chain for short chains, the observed absolute effects (Figure 3a) are less prominent. Normalizing the corresponding results (Figure 3b) reveals that the relative changes are on a very similar level.

Please note that our initial value of the number of entanglements *Z* and hence entanglement length $N_{e}$ are on the outer edge of experimental results, as e.g., discussed in [40].

### 3.2. System State after Cooling Using Fixed Conditions

**Figure 4:**
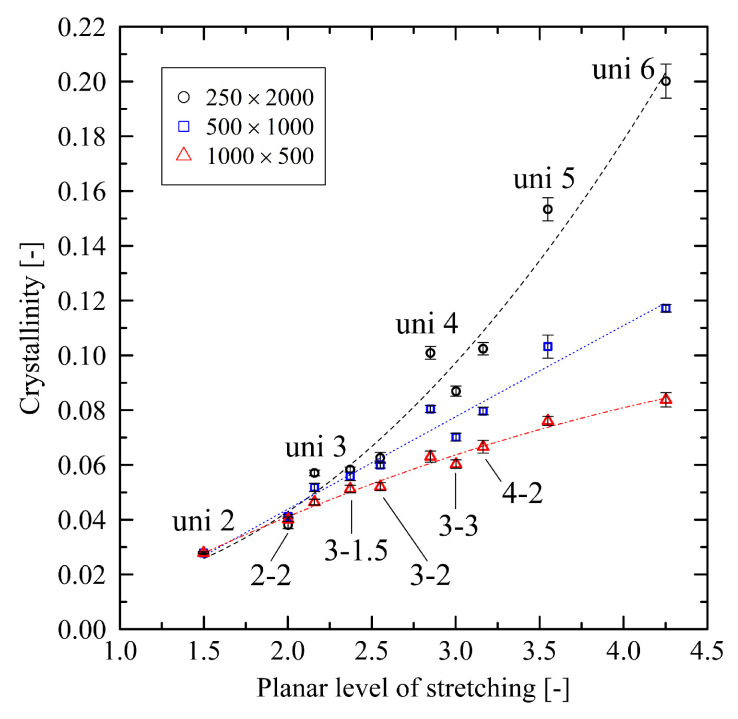
Crystallinity plotted over the planar level of stretching for different system sizes (M×N). Inserts denote the levels of stretching for the corresponding data points. The dotted lines represent second-order polynomial fits for each system size. They are a guide to the eye only.

To investigate the relation between the internal structure after stretching and subsequent crystallization behavior, we initialize the crystallization process by cooling the systems under fixed conditions from 500 K to 293.15 K (cooling time 10 ns). By evaluating the degree of crystallinity immediately after cooling, we see that an increasing level of stretching results in an increasing degree of crystallinity after cooling (Figure 4). Results show a significant dependency on the chain length: For the shortest investigated chain length (N=500), we monitor a decreasing slope of crystallinity with increasing level of stretching, while for the longest chain length (N=2000) there is a clearly rising slope. The system with chain length N=1000 falls in between these results. Again, the main stretching direction plays a dominating role. The resulting level of crystallinity for the uni 4 and 4-2 system in particular are very close to each other for all system sizes.

**Figure 5:**
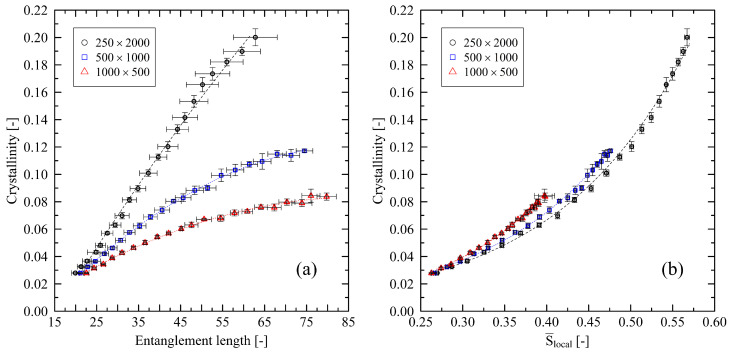
Crystallinity plotted over entanglement length $N_{e}$ (**a**) and nematic order parameter ${\bar{S}}_{\mathrm{local}}$ (**b**) for different system sizes (M×N). All systems are uniaxially stretched to levels of stretching in the range $2\leq \lambda_{\mathrm{uni}}\leq 6$ (step size 0.2). The dotted lines represent second-order polynomial (**a**) and exponential fits (**b**), respectively. They are a guide to the eye only.

Results from Figure 4 pose the question of how crystallization is micro-mechanically supported after stretching. Hence, we compare the entanglement length ($N_{e}$) and the level of nematic ordering (${\bar{S}}_{\mathrm{local}}$) after stretching with the resulting level of crystallinity after cooling (Figure 5). We first start by investigating uniaxially stretched systems only.

Clearly, a correlation of the entanglement length after stretching and the resulting level of crystallinity exists (Figure 5a). More extensive entanglement lengths (less entangled chains) allow the chains to reach a higher level of crystallization. Interestingly, at the same level of entanglements, long chains have a significantly higher level of crystallization than short chains.

Taking the nematic ordering into account (Figure 5b), we report that strong initial local ordering of chain segments results in an increasing level of crystallinity. At the same level of nematic ordering, there is only a minor difference between different chain lengths with respect to crystallinity. The main difference here is the fact that longer chains are able to align to well-ordered structures to an increasing extend (${\bar{S}}_{N=500,\lambda =6}=0.39\pm 0.01$; ${\bar{S}}_{N=1000,\lambda =6}=0.48\pm 0.01$; ${\bar{S}}_{N=2000,\lambda =6}=0.57\pm 0.01$). This also explains the indicated saturation behavior in Figure 5a: Strongly stretched systems with short chain length are limited concerning the formation of well-ordered structures due to stretching, which results in lower levels of initial crystallinity.

Combining these findings, we conclude that the number of entanglements per chain is a crucial factor that strongly impacts initial crystalline structures after stretching. Only well-entangled long-chain systems are capable of forming strongly structured local areas. This supports our finding that short chains tend to be pulled apart as a whole, whereas locally ordered structures are built up between entanglement points (cf. [24]).

Additionally, Figure 5a illustrates that for the longest investigated chain length (N=2000, which according to our coarse-grained model represents 6000 repeating units) the relation of entanglement length and crystallinity is almost linear. Systems with shorter chain lengths follow a second-order polynomial slope. As real-life polymers used in industrial application have chain lengths beyond 6000 repeating units, we state that the decreasing slope for the short-chain systems shows that these are not suitable for investigations at large levels of stretching. In addition to our results in [24], we can reach a limit from which short-chain systems start not to represent the behavior of long chains. By assuming a linear regime (with coefficient of determination $R^{2}\geq 0.99$) at the beginning of each curve, we give an estimate of the point from which non-linear behavior dominates. From the mean values as plotted in Figure 5a, we calculate $N_{e,\mathrm{limit}}=38$ and 52 for chain length 500 and 1000, respectively. These values correspond to a uniaxial level of stretching of $\lambda_{\mathrm{uni},\mathrm{limit}}=3.3$ and 4.6. For the system with a chain length 2000, we can extrapolate our results by using the corresponding polynomial fit. Calculation predicts $N_{e,\mathrm{limit}}=72$ ($\lambda_{\mathrm{uni},\mathrm{limit}}=6.5$).

**Figure 6:**
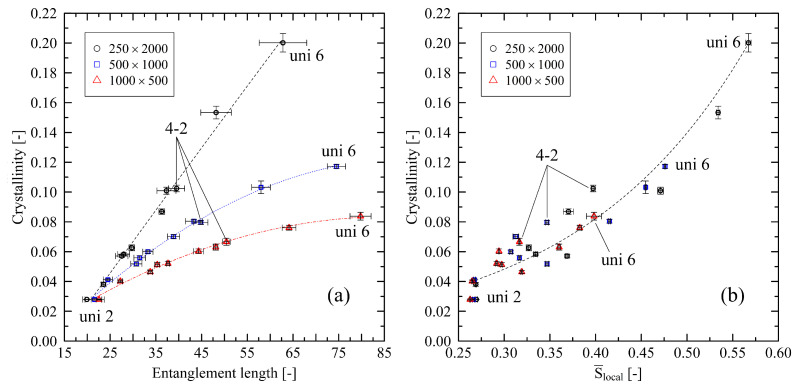
Crystallinity plotted over entanglement length $N_{e}$ (**a**) and nematic order parameter ${\bar{S}}_{\mathrm{local}}$ (**b**) for different system sizes (M×N). The figure includes data points for all biaxially stretched systems (2-2; 3-1.5; 3-2; 3-3; 4-2) and uniaxially stretched systems (uni 2; uni 3; uni 4; uni 5; uni 6). The dotted lines represent second-order polynomial (**a**) and exponential fits (**b**), respectively. They are a guide to the eye only.

Considering biaxially stretched systems, Figure 6a reveals that in the case of the relationship between entanglement length and degree of crystallinity results fall in line with the purely uniaxial investigations from Figure 5. In addition, it can be noted that entanglement length and crystal size are associated with each other. An increase in the entanglement length leads to larger average crystal sizes. For example, in cases of the 250×2000 system, average crystal size (determined according to our methods described in [24]) increases almost linearly by a factor of 1.40±0.03 from the lowest (uni 2) to the largest (uni 6) investigated level of stretching. This also holds for the investigations under free conditions (cf. Section 3.3) but with a lower increase in the crystal size by a factor of 1.16±0.01.

Concerning the nematic ordering (Figure 6b), results do not allow one to properly distinguish results for different chain lengths. Moreover, the influence of different biaxial stretchings does not strictly fall in line with the uniaxial data points. Hence, we can only give a general estimation for the expected trend, fitted over all data points.

### 3.3. System State after Cooling Using Free Conditions

**Figure 7:**
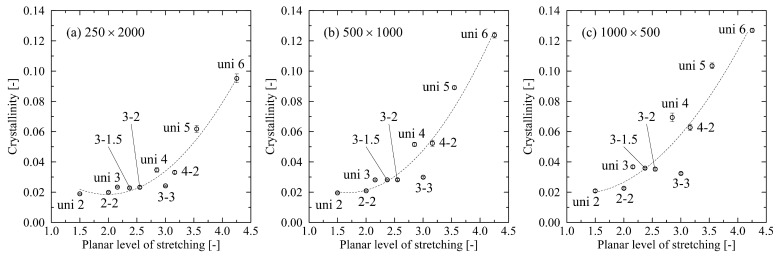
Crystallinity plotted over the planar level of stretching for different system sizes (M×N). (**a**) system size: 250×2000, (**b**) system size: 500×1000, (**c**) system size: 1000×500. Inserts denote the levels of stretching for the corresponding data points. The dotted lines represent second-order polynomial fits for each system size. They are a guide to the eye only.

In this subsection, we compare the influence of fixed and free boundary conditions on the results. Figure 7 gives insight into the relation of the planar level of stretching and the resulting level of crystallinity for systems which were stretched under free conditions. In contrast to Figure 4, we monitor increasing slopes across all investigated chain lengths. Moreover, the level of crystallinity now decreases with increasing chain length.

**Figure 8:**
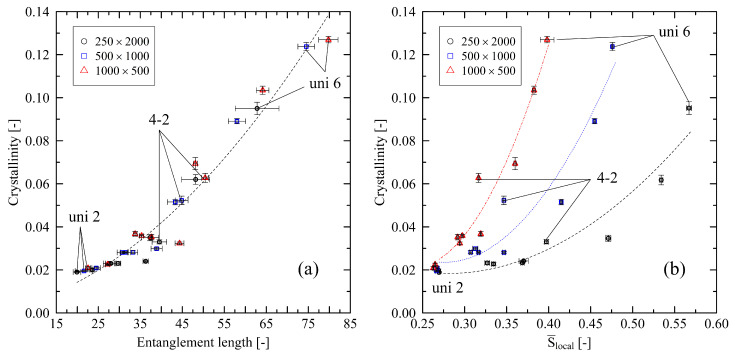
Crystallinity plotted over entanglement length $N_{e}$ (**a**) and nematic order parameter ${\bar{S}}_{\mathrm{local}}$ (**b**) for different system sizes (M×N). The figure includes data points for all biaxially stretched systems (2-2; 3-1.5; 3-2; 3-3; 4-2) and uniaxially stretched systems (uni 2; uni 3; uni 4; uni 5; uni 6). The dotted lines represent second-order polynomial fits. They are a guide to the eye only.

**Figure 9:**
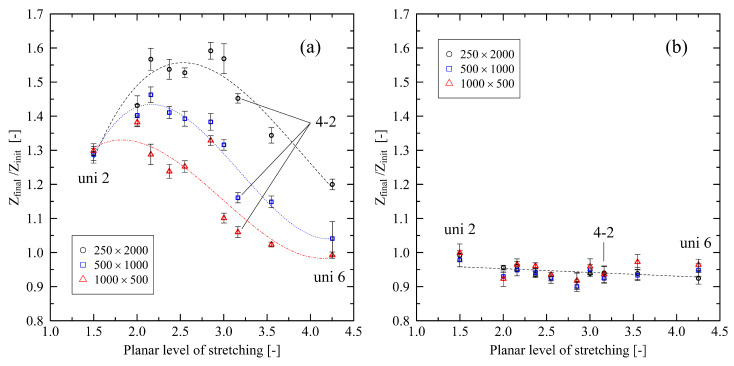
Ratio of number of entanglements per chain *Z* at the end ($Z_{\mathrm{final}}$) and at the beginning of the cooling stage ($Z_{\mathrm{init}}$) for different system sizes (M×N). Data are plotted over the planar level of stretching. (**a**) Results for free conditions, (**b**) results for fixed conditions. The figures include data points for all biaxially stretched systems (2-2; 3-1.5; 3-2; 3-3; 4-2) and uniaxially stretched systems (uni 2; uni 3; uni 4; uni 5; uni 6). The dotted lines in (a) represent third-order polynomial fits for each investigated chain length. In (b) the dotted line is a linear fit over all data points. All fits are a guide to the eye only.

By investigating the micro-mechanical structure of the stretched polymer systems, Figure 8a shows that the entanglement length influences the resulting level of crystallinity. In contrast to the results in Section 3.2, the relation of entanglement length and level of crystallinity is independent of the chain length. This behavior hints towards strongly rebuilding entanglements after immediate release of the systems that is stronger for longer chains (cf. [24]). Figure 9a supports this finding by showing that the ratio of the number of entanglements per chain *Z* at the end of the cooling stage ($Z_{\mathrm{final}}$) and at the beginning of the cooling stage ($Z_{\mathrm{init}}$) increases with increasing chain length. That effect makes results in Figure 8a indistinguishable for different chain lengths. On the other hand, under free conditions, the relevance of local chain ordering becomes the dominating factor with respect to the level of crystallization after cooling (Figure 8b). We want to point out that in systems consisting of shorter chains, a low initial level of nematic ordering is sufficient to induce rapidly increasing levels of crystallinity. Again, this falls in line with Figure 9a, which shows that especially short chains at high levels of stretching do not or only slightly refold ($Z_{\mathrm{final}}/Z_{\mathrm{init}}\approx 1.0$) after release. This indicates that during stretching short chains tend to be pulled apart as whole from each other. That behavior leads to a greater conservation of local crystalline structures (i.e., higher level of crystallinity) in contrast to longer, strongly refolding chains (cf. Figure 7).

Figure 9a also reveals that for different chain lengths a peak region with respect to their individual potential to refold exists. For comparison, results for the fixed conditions in Figure 9b show that unfolding of chain segments during cooling slightly increases with increasing level of stretching.

## 4. Discussion

As expected, analyzing uni- and biaxially stretched systems reveals strong dependencies on the chain length. As we have already shown in [24], there is significantly more orientation in systems consisting of long chains (cf. Figure 2). We can now augment previous results by reporting that the nematic ordering of chain segments predominantly depends on the major biaxial stretching direction. This finding is supported by the investigations in [25], where they show that ordering of the chain end-to-end vector increases with increasing stretch ratios. This falls precisely in line with our exploration of the uniaxial factor in Figure 1. Extending the results from [25], we can demonstrate that at a fixed stretch ratio, an increasing trend of orientations for higher stretch ratios exists (Figure 1, systems: uni 2; 3-1.5; 4-2).

After cooling the stretched systems below crystallization onset temperature, we monitor two different situations: In the case of the fixed conditions, there is an increasing level of crystallinity with increasing chain length. For the free conditions, relations are reversed. The main differences between these specific cases are re-entangling effects, which have a crucial impact on the crystallization behavior (cf. Figure 9). This supports our findings in [24], where we already revealed that in the case of free conditions, re-entangling effects play the dominating role concerning initial crystallization behavior. Generally, we see higher amounts of crystallinity at the end of the cooling procedure for systems with a high level of local nematic ordering (cf. Figure 6 and Figure 8). Again, this is in line with [25], who reports faster growth of crystalline clusters in biaxially stretched systems with increasing local orientation after stretching.

Finally, our models indicate that the relation of the entanglement length after stretching and resulting crystallinity after cooling (cf. Figure 6) considerably depends on the chain length: we show that short-chain systems (N=500,1000), in contrast to systems with long chain length (N=2000), exhibit a remarkable non-linear relationship. As demonstrated in [24] this behavior is due to pulling short chains apart as a whole at higher levels of stretching. This effect inhibits such systems from the formation of larger crystalline areas.

Generally, our observations of the combination of disentanglement and the resulting greater orientation and crystal nucleation is in agreement with other simulation studies ([19,20,25]). Comparing our specific results with experimental observations is highly demanding as the investigated initial crystallization phase during fast cooling is experimentally inaccessible. The fastest experimental cooling rates are in the range of 1200 K/s [41]. Nevertheless, trends towards a stretching-induced increase in crystallization as well as orientation-dependent properties are reported in different experimental studies with focus on different polymer processing conditions (blow-molding [6], film blowing [42]). These experimental results as well as our simulation results are in agreement with expectations from flow-induced crystallization (FIC) theory [22,43,44,45].

## 5. Summary

Our study analyzed the micro-mechanical states after uni- and biaxial stretching procedures for polyethylene systems. Two different approaches were used: (a) “free” conditions, which allow the systems to contract instantaneously after stretching and (b) “fixed” conditions, which hold the box dimensions fixed during solidification of the melt. Both procedures represent loading conditions that occur in real-life polymer processing.

By using large systems with chain lengths up to N=2000 beads, we can realistically model effects that are relevant on the macro scale. First, analysing the initial structure after stretching reveals that, in many cases, the main stretching direction dominates the micro-mechanical states: Nematic ordering and the entanglement state show distinct dependencies from the major biaxial stretching direction. Beyond that, we are able to demonstrate specific cases where biaxial stretching has a negative impact on the local ordering of chain segments and, in consequence, crystallinity.

A deeper analysis of the crystallization behavior after cooling of the differently stretched systems gives substantially different results for the free and fixed boundary conditions: For the initial crystallization behavior, while cooling, the entanglement length and re-entangling effects play the dominating role, whereas nematic ordering is of minor importance. In the case of fixed boundary condition, this results in high levels of crystallinity for strongly entangled systems (N=2000). In contrast, the use of free conditions enforces low levels of crystallinity in strongly entangled systems due to re-entangling effects.

Finally, our investigations clearly show the impact of chain length on the results. For each investigated chain length, we demonstrate that a specific level of stretching exists, from which chains do not reflect real-life polymer behavior.

In this study, we investigated system states immediately after cooling. In a future study, we will explore how these systems develop during further relaxation processes.

## Figures and Tables

**Table 1:** Bonded and Lennard–Jones force-field parameters for coarse-grained polyethylene [28]. Note that the Lennard–Jones parameters depend on the particle position.

Bead Type	Bond Length		Bond Angle		Dihedral Angle	
	$b_{0}$	$K_{b}$	$\theta_{0}$	$K_{\theta }$	m	$K_{\phi }$
	**(nm)**	**(kJ mol${}^{-1}$ nm${}^{-4}$)**	**(${}^{\circ }$)**	**(kJ mol${}^{-1}$)**	**(-)**	**(kJ mol${}^{-1}$)**
CG3	0.353	19730	146.4	56.6	1	0.74
**Bead Type**	**Position**	σ	ϵ	$\sigma_{1-4}$	$\epsilon_{1-4}$	
		**(nm)**	**(kJ mol${}^{-1}$)**	**(nm)**	**(kJ mol${}^{-1}$)**	
CG3_mid_	middle	0.457	2.214	0.401	2.213	
CG3_end_	end	0.468	2.415	0.421	2.415	

## Data Availability

The data that support the findings of this study are available from the corresponding author upon reasonable request.
